# How green are the streets? An analysis for central areas of Chinese cities using Tencent Street View

**DOI:** 10.1371/journal.pone.0171110

**Published:** 2017-02-14

**Authors:** Ying Long, Liu Liu

**Affiliations:** 1 School of Architecture and Hang Lung Center for Real Estate, Tsinghua University, Beijing, China; 2 China Academy of Urban Planning and Design, Shanghai, China; Beihang University, CHINA

## Abstract

Extensive evidence has revealed that street greenery, as a quality-of-life component, is important for oxygen production, pollutant absorption, and urban heat island effect mitigation. Determining how green our streets are has always been difficult given the time and money consumed using conventional methods. This study proposes an automatic method using an emerging online street-view service to address this issue. This method was used to analyze street greenery in the central areas (28.3 km^2^ each) of 245 major Chinese cities; this differs from previous studies, which have investigated small areas in a given city. Such a city-system-level study enabled us to detect potential universal laws governing street greenery as well as the impact factors. We collected over one million Tencent Street View pictures and calculated the green view index for each picture. We found the following rules: (1) longer streets in more economically developed and highly administrated cities tended to be greener; (2) cities in western China tend to have greener streets; and (3) the aggregated green view indices at the municipal level match with the approved National Garden Cities of China. These findings can prove useful for drafting more appropriate policies regarding planning and engineering practices for street greenery.

## 1. Introduction

As one of the most prominent colors in nature, green has been an everlasting beloved color of humans, and the “garden city” advocated by [1] is among the most famous planning theories. According to [2], green spaces offer significant potential for restoration, correspond to the innate human tendency to focus on life and lifelike processes, and promote behaviors that boost well-being; thus, increasing the provision and utilization of urban green spaces can promote stress reduction, happiness, health, and well-being among humans. As an essential aspect of green-city implementation, green coverage at various scales—at the block level (green land area divided by block area), for example, or citywide (total green land area divided by the city’s urban land area)—is a mandatory element of spatial plans for promoting a high quality of life. As a result of partial planning implementation and the diverse composition of green spaces, green coverage in planning drawings does not directly correspond to the total greenery in reality. This is one reason why visual greenery has been extensively discussed in the research community and is suggested for use in practice. Although not required in spatial plans, street greenery—as the focus of this study and a key indicator for evaluating urban form at the city-design level—is important for citizens’ quality of life (especially for pedestrians in daily life); however, this has not been sufficiently studied due to a lack of fine-scale data.

Understanding how green our streets are has never been easy. Using the conventional methods, it is generally time consuming and expensive. To address this issue, we developed an automatic method using a street-view service while also borrowing and modifying ideas from existing studies such as [3–5]. The green color ratio in street views (termed “green view index” in this paper)—which reflects objective city (as well as rural in most street-view products) street (and road) landscapes—was selected as the proxy for linking with street greenery in this study, which falls under the umbrella of visual greenery studies. Different from online geotagged photos, which reflect city images captured subjectively by photographers, street view objectively depicts the true urban landscape. This is another reason why we chose street view to understand street visual greenery in this study. Today in China, academic studies are increasingly using open data from social networks, commercial websites, and official channels to understand city systems and urban structure, as well as human mobility and activity (see [6] for a review). To the best of our knowledge, this is one of the first studies to analyze street greenery in a large number of cities using street view.

This paper is organized as follows: To illustrate the research context, section 2 reviews related areas such as visual greenery and using street-view pictures for urban studies. Sections 3 and 4 introduce the study area, data, and research methods. Section 5 presents the research results in various aspects—such as the overall pattern, intercity rankings and analysis, and intracity pattern analysis—as well as the validation of the results. In the final section, we discuss potential applications, academic contribution, research biases, and future plans.

## 2. Literature review

### 2.1 Using street-view pictures in urban studies

Systems like Google Street View and Bing Maps Streetside enable users to virtually visit cities (on the streets or even indoors) by navigating immersive 360° panoramas. There are various endeavors related to Google Street View (GSV) image recognition, including 3-D city model construction [7], commercial-entity identification [8], real-time text localization and recognition [9], and layer interpretation for ground, pedestrians, vehicles, buildings, and sky [10].

In addition to these existing studies in the field of computer science, there are related studies in urban geography, regional science, urban studies, and urban planning. Rundle et al. [11] suggest that GSV can be used to audit neighborhood environments by checking the concordance between GSV analysis and field surveys. Odgers et al. [12] observed children’s neighborhoods using GSV and found it to be a reliable and cost-effective tool. Kelly et al. [13] used GSV to audit built environments and also found it to be a reliable method. Hwang and Sampson [14] identified visible clues of neighborhood gentrification using GSV for systematic social observation. Carrasco-Hernandez [15] reconstructed building geometries and urban sky view factors using the GSV image database. In general, street view has proven to be an effective and reliable tool for measuring built environments on various scales, such as streets and neighborhoods. The aforementioned studies were all conducted manually by looking at street-view images, not by automatic means. This time-consuming process places constraints on using street view to analyze large geographical areas. We did find an investigation [16] that combined crowdsourcing techniques with GSV to identify street-level accessibility problems, but this still relied heavily on manual human effort.

Based on our review of the use of street view in two general fields (computer science and urban studies), we found the following mismatch. Computer scientists have been developing advanced image recognition algorithms to automatically identify specific objects, texts, or patterns from street view. Urban scientists, however, have employed street view manually, without drawing on the latest progress made by computer scientists. Such time-consuming techniques are not easy for urban scientists to overcome. The second author of this paper has proposed a solution that involves automatic cognitive city mapping using geotagged photos (not street-view pictures), drawing on Kevin Lynch’s *The Image of the City* [17–18]. The present study aims to further explore using street-view pictures to automatically and exhaustively analyze/visualize street greenery in our cities, and thus contribute to building a science of cities [19].

### 2.2 Understanding visual greenery

The effort to bring natural greenery into urban environments has a long history. In the 1850s, Olmsted focused on urban park reform and street design, trying to combine natural environments with urban living spaces [20]. The greenway movement in the late 1980s was a large-scale concept that proposed creating a green network to give people access to open spaces close to where they live and to link rural and urban spaces in the American landscape [21]. Such urban “green constructions” are mostly valued for their economic or environmental benefits. A study of the cooling effects of street greenery at 11 urban sites in Tel-Aviv showed that the shaded area under a canopy plays a key role in alleviating the “heat island” effect [22]. Another important contribution of street-level vegetation is that it improves air quality along street canyons, which has been studied by many researchers like [23].

However, aspects of the visual effect or aesthetic amenity of greenery have received less attention. The ratio of greenery as a measurement of the visibility of street greenery, first proposed by [24], calibrates the effective ratio for a variety of landscape scenes via different focal distances. Ohno [25] further studied the measurement of ambient visual information. He suggested that while visible greenery can soften the negative impression of an “artificial” environment, the positive impression of a “natural” environment is not enhanced when the ratio exceeds 15%. In a later study, Ohno [26] emphasized auto-centricity in vision, which relates to people’s feelings and their experience of pleasure. Nasar [27] investigated visual environment preferences for urban street scenes among 46 students from Japan and the US. The preference scores showed that students from both countries preferred foreign scenes to native ones and that preference was associated with the prominence of nature, and the absence of vehicles.

Photos have been regarded as promising data sources for measuring visual greenery. Three methods should be noted. **First**, Yang et al. [3] developed the green view index to evaluate the visibility of urban forests through a combination of field surveys and manual photograph interpretation. They found a strong correlation between the green view index and the canopy cover of trees/shrubs. This method relies heavily on human labor. **Second**, Google Street View provides opportunities for automatically gathering a large number of street pictures. Li et al. [4] proposed an automatic framework for assessing street-level urban greenery using GSV and employed it to study the East Village in Manhattan. The quality of this methodology has been validated through manually derived street greenery in Photoshop. The identification method for street greenery is pixel-based color recognition. Li et al. [5] further analyzed the relationship between automatically calculated street greenery using GSV and residents’ socioeconomic characteristics in Hartford, Connecticut. Li et al. [28] also explored environmental inequities among different types of urban greenery; since automatically recognizing street greenery using street-view pictures is time consuming, the study area was small. **Third**, another method for understanding visual greenery is pattern recognition using semantic pixel-wise segmentation and scene labeling. Group labeling based on a combination of RGB- and depth-based cues could obtain higher accuracy, according to several experimental studies. Farabet et al. [29] achieved real-time scene parsing and image labeling to best explain the scene. Gupta et al. [30] recognized indoor scenes using boundary detection and hierarchical grouping. By adding global appearance features, Gatta and Romero [31] conducted spatial scene parsing. Badrinarayanan et al. [32] developed SegNet for more accurate outdoor and indoor element recognition, by which 11 classes, including trees, were labeled. Compared to the pixel-based color-recognition approach, this approach requires more time and techniques.

Based on our review of these three methods, we used the approach developed by Li, with modifications, to study a large number of cities in China, aiming to gain a holistic understanding of street greenery at the city-system level. A detailed comparison of Li’s method and ours is discussed in Section 6.3.

## 3. Study area and data

### 3.1 Study area

**Fig 1:**
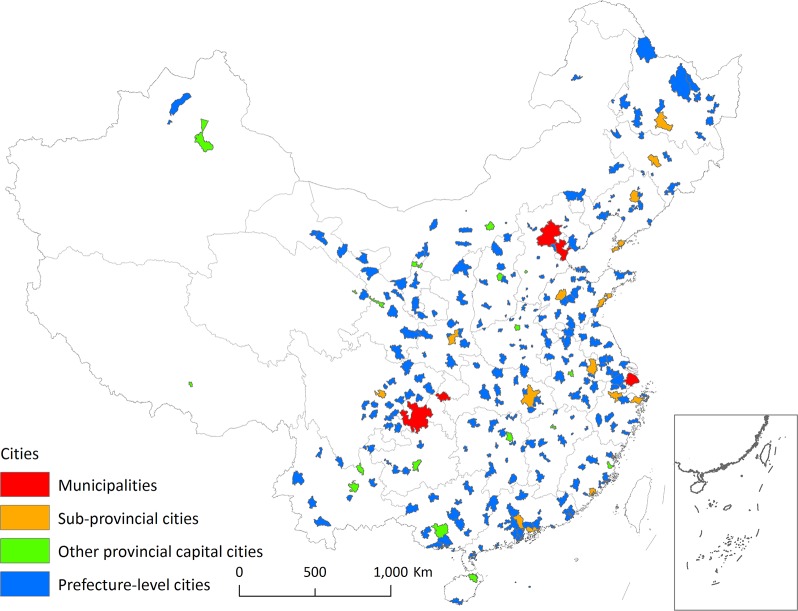
The 245 cities at or above the prefecture level with street-view service in China.

In China, there are 288 cities at or above the prefecture level. There are 5 administrative levels in China, and county-level cities were not included in this study. Since 43 of the cities did not have street-view data, our study included 245 cities. Among these, there are 4 municipalities directly governed by the central government, 15 subprovincial cities, 17 other provincial capital cities, and 209 prefecture-level cities (Fig 1). Considering the crawling, processing, and computation load of street-view pictures (SVPs), we only focused on the downtown areas of each city. Since the downtown or live-work-play center boundaries are not available for most Chinese cities, we buffered each city center (namely, the central business district [CBD]) with a radius of 3 km to derive the study areas. These are regarded as proxies for China’s CBDs and have a total area of 8,143 km^2^. We acknowledge potential bias in this translation process and will address it in future studies.

### 3.2 China’s roads/streets in 2014

**Fig 2:**
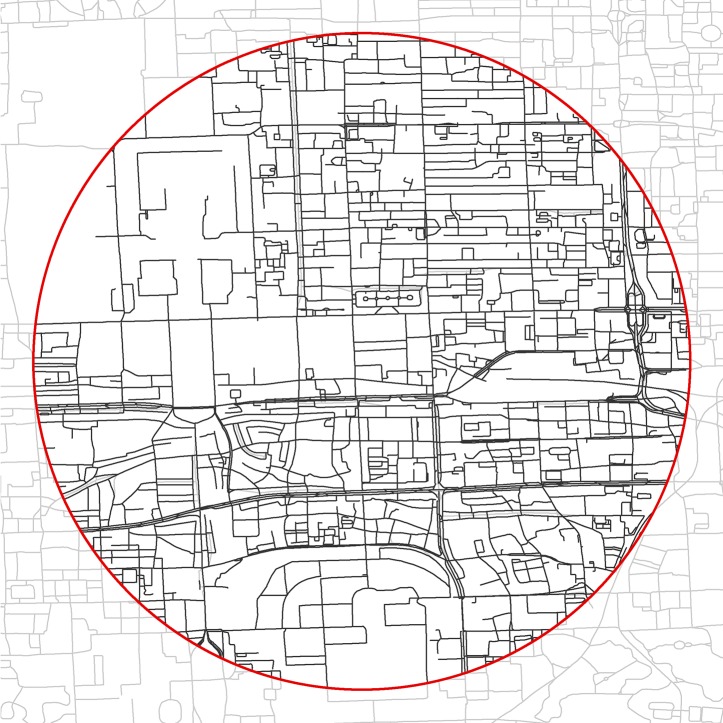
Roads/streets of the study area in Beijing in 2014.

We used the 2014 roads/streets of China obtained from a local road-navigation firm based in Beijing for crawling SVPs. Almost all detailed road/street networks at various levels, including streets and regional roads, are included in this dataset as of the end of 2014, according to comparisons with Google Maps and Baidu Maps (a main online map service provider and popular search engine in China; http://map.baidu.com). The total road/street length is 4,249,419 km for 11,562,709 street segments (367.5 m in average). Note that a street segment denotes a part or all of a street divided by intersections. We clipped the national dataset with the study area (245 polygons in a circle) and derived the street segments within the study area, including 748,430 street segments with a total length of 66,041 km (88.2 m in average). As shown in Fig 2, some of the raw streets are dual lines or bridges, which made it necessary to preprocess the data for crawling SVPs. We also removed highways and bridges to avoid the street-level images captured in them, which are not suitable for studying street greenery. After the data preprocessing, there were 372,186 street segments with a total length of 54,180 km. This process is explained in section 4.1.

## 4. Methodology

**Fig 3:**
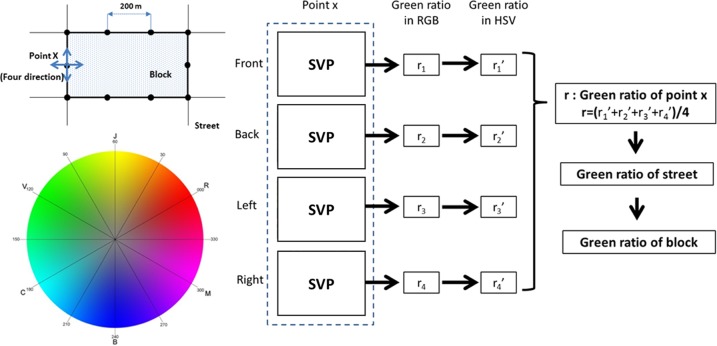
Framework for analyzing street greenery in a typical area (SVP = street view picture).

The process for evaluating street greenery involved four steps: street data preprocessing, data crawling, picture analysis, and results aggregation (Fig 3). The details of each step are as follows.

### 4.1 Simplifying street segments for better crawling SVPs

**Fig 4:**
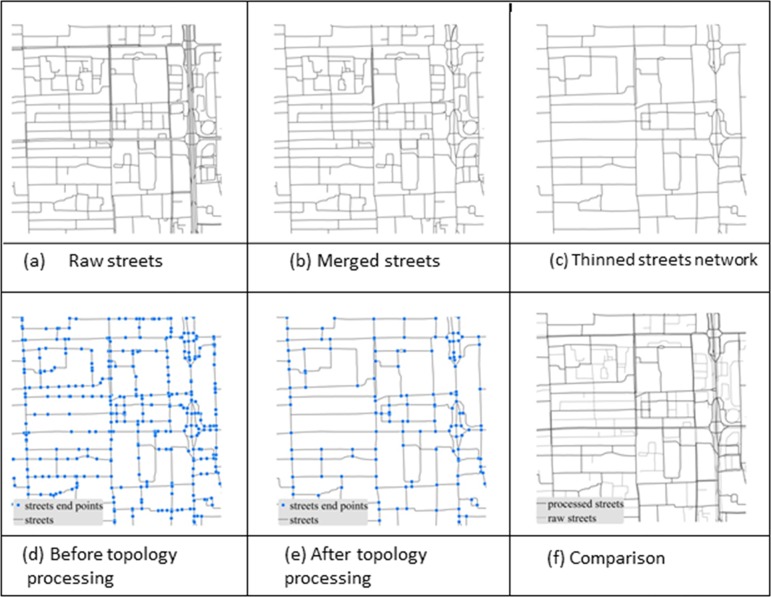
Process for simplifying street segments.

The raw streets were simplified and generalized using Esri’s ArcGIS (Fig 4). We first merged them into single-line street features using the “merge divided roads” tool. Matched pairs of streets or lanes were merged if they were in the same road class, generally parallel to each other, and within merging distance of each other (Fig 4B). We then used the “thin road network” tool to generate a simplified street network that retained the connectivity and general characteristics of the merged streets (Fig 4C). After that, “must not have dangles,” “must not have pseudo nodes,” and “must not intersect” were added as topology rules to delete dangling roads, delete pseudo nodes, and avoid breaking streets into segments at intersections (Fig 4E).

### 4.2 Crawling SVPs from Tencent Maps

In China, Google Street View–like services have been introduced by various online map providers, including Baidu, Netease, Amap (Gaode), and Tencent. Among these, Tencent’s street view (http://map.qq.com) has the largest coverage. According to Tencent’s website (http://map.qq.com/jiejing/home.html; accessed September 6, 2015), almost all of the accessible streets in 272 covered cities are associated with high-quality SVPs, and most regional roads and hot spots, like tourist areas, are covered. The experience of using Tencent Street View is quite similar to using GSV.

Tencent Maps provides an application programming interface (API) for querying and downloading SVPs. The parameters required by the API are listed in Table 1, which include the SVP size, the location or place ID, horizontal and vertical angles, and the developer’s key. Detailed guidelines for downloading SVPs are available online (http://lbs.qq.com/panostatic_v1/guide-getImage.html). The query frequency is limited to 100,000 queries per key. A demo of the API is available at http://apis.map.qq.com/ws/streetview/v1/image?size=600x480&pano=10011022120723095812200&pitch=0&heading=0&key=OB4BZ-D4W3U-B7VVO-4PJWW-6TKDJ-WPB77. After consulting the website and checking Tencent’s data policy, we confirmed that we were compliant with Tencent’s terms of service. Since it is not guaranteed that each queried location/Pano (see Table 1) will be associated with SVPs, Tencent provides other APIs that retrieve place IDs by querying a coordinate’s neighboring area. Details are available online at http://lbs.qq.com/webservice_v1/guide-adsorb.html. A place ID can be returned for a coordinate within a predefined search radius, if one exists (in some cases, there are no SVPs at the location due to the availability of street-view service).

**Table 1:** Parameters for the SVP crawling API.

Parameter	Mandatory item?	Description	Example
Size	Yes	Picture size in pixels; maximum width and height: 960 by 640	Size = 138 x 187
Location	Either location or pano	Coordinates or place name for confirming the street-view location	Location = Tsinghua University or location = 39.12,116.83
Pano		Street-view ID for confirming the street-view location	Pano = 10011022120723095812200
Heading	No	The angle of the forward direction in relation to the north, measured clockwise from 0 to 360 degrees (0 is the default value)	• North: heading = 0 • East: heading = 90 • South: heading = 180 • West: heading = 270
Pitch	No	The vertical angle the camera covers, -20 to 90 degrees, in which a positive number indicates the degree of looking up and vice versa (0 is the default value)	Pitch = 0
Key	Yes	Developer’s key (can be retrieved through online application)	Key = OB4BZ-D4W3U-7BVVO-4PJWW-6TKDJ-WPB77

We first divided each processed street segment into vertices with a distance of 50 m, and each vertex’s coordinates were input into the place ID retrieval API and the main API for downloading SVPs. The sampling distance could lead to incomplete coverage in cities. This compromise was also adopted by Li et al. [4], who randomly selected sample sites along streets, and the shortest distance allowed between any two randomly placed points was set to 30 m. We set the size of each retrieved SVP to 960 by 640 pixels, and the vertical angle (*pitch* in the API) was set to 0 degrees. We did not consider the vertical angle in this study, reserving it for future research. For each vertex, we queried the API four times with different horizontal angles (*heading* in the API), including the front, back, left, and right sides. This was enabled by calculating the direction of each street segment further divided by the adjacent two vertices. Finally, all SVPs for the study area were then retrieved, along with a table showing the inventory of all SVPs, including the necessary information (e.g., coordinates, street segment ID, horizontal angle, SVP file name, and horizontal angle).

### 4.3 Analyzing the color composition of crawled SVPs

As another core procedure, we analyzed the color composition using MATLAB. For each photo, we converted its format from RGB to HSV and then extracted the value of the hue channel from the digital image. The pixel ratio calculation was then divided into 360 bins, indicating the percentage of each degree shown in the color spectrum (Fig 3). The summarized result of all 360 attributes is 1 for each SVP. Based on a careful review of the color spectrum, we defined a color range of 60 to 180 as greenness. This color range was confirmed by benchmarking our GVI calculation results with the “ground truth,” which was interpreted manually using Photoshop. We randomly selected 100 SVPs from our samples. We then manually calculated the GVI for the 100 SVPs in Photoshop using the same method as [4]. The correlation between the Photoshop-derived GVI values and those obtained through our study method reached a maximum level (0.87) when we used a green color range of 60–180, thus justifying our parameter selection. Different illumination conditions or shadows could have influenced the calculation results; this will be addressed in future studies. In this way, we calculated the greenness ratio for each SVP by summing the attributes falling into the range of 60–180. We acknowledge potential bias in setting the greenness range, which will be addressed in future studies. Since there are generally four SVPs at a street vertex (front, back, left, and right), we averaged the greenness percentage as the green view index (GVI) of each street vertex. The GVI used here is similar to that of [4]. We then classified the GVI into four categories—not green (≤ 0.2), somewhat green (0.2–0.4), green (0.4–0.5), and very green (> 0.5)—which are used in the following mapping and analysis.

We used the GVI as a proxy for street greenery. Ideally, we wanted to identify urban greenery (e.g., trees, shrubs, grass) via color recognition. We acknowledge that this method presents a problem with distinguishing street greenery from other green objects (e.g., cars, logos, or even walls). This can be alleviated by using an image segmentation approach in future research.

### 4.4 Aggregating point-level GVI to street and block levels

To clearly visualize and analyze the GVI calculation results, we needed to aggregate the vertex-based GVI to the street and block levels. Each vertex was associated with a street segment, and the average and standard deviation of the GVI could be directly calculated, along with the total vertices of the street segment. The point-level GVI can be aggregated to the administrative region or ZIP code level as well, according to different research purposes.

To assign the street-level GVI to blocks, we first needed to generate blocks. For this study, a block was defined as a continuous polygonal area confined by streets. We delineated the street space, and individual blocks were formed as polygons bounded by streets. Based on our previous study [33], the delineation of street space and blocks was performed as follows: (1) All processed streets were merged as line features in a single data layer. (2) Street spaces were generated as buffer zones around street networks; a varying threshold ranging from 2 to 30 m was adopted for different street types (e.g., surface condition as well as different levels of streets). (3) A block was delineated as the space left when the street space was removed. Given the nature of delineated blocks, most GVI locations/sites on streets were not in any blocks. We then averaged the GVI of the locations surrounding the block as the GVI of the block, which was enabled by assigning each GVI location to its closest delineated block.

## 5. Results

### 5.1 Profile of crawled SVPs

There were 336,990 locations/sites with crawled SVPs. The average GVI was 0.248 for all locations. To demonstrate our SVPs and their calculation results, we shared the Beijing street-level GVI analysis results online at http://geohey.com/apps/dataviz/576beaa309af4f589420c837829fb6c9/share?ak=ZmYzNmY0ZWJhYjcwNGU2ZGExNDgxMWUxNmZiOWNhNGY. Note that this online map covers a much larger area of Beijing, not limited to the downtown area.

We noticed that some cities with less green were associated with street views taken in autumn or winter. Such seasonal differences in the photos could significantly influence the vegetation extraction and produce less convincing results. Unlike Google Street View, the time stamps of the SVPs are not obtainable through the API provided by Tencent, thus making it more difficult to use SVPs to evaluate street greenery in China. Although time stamps are not available via API, they are printed on each picture, enabling us to manually examine the season issue. We identified 114 cities (including all municipalities) with SVPs taken during the fall or winter. Thus, we focused on the remaining 131 cities, which had 163,853 street segments and 173,425 locations in total.

### 5.2 Intercity ranking and analysis

**Fig 5:**
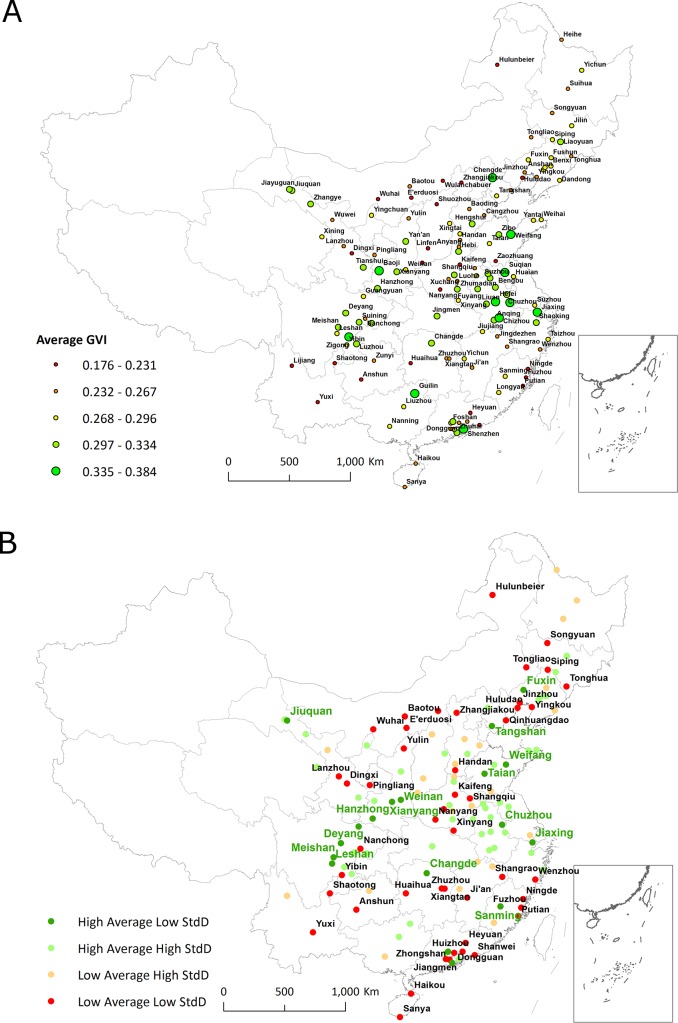
Spatial distribution of the average street GVI of each city (a) and the four types of cities classified (b).

We ranked the remaining 131 cities in terms of the average GVI for all sample points (Fig 5A) and found that the average GVI ranged from 0.132 to 0.384 at the city level. It should be mentioned that street-view greenness could be affected by the width of a street. Street width can vary in different cities, and we will devote more attention to adjusting the calculation results in future studies. The top five greenest downtowns were Weifang, Zigong, Baoji, Maanshan, and Chengde; the bottom five were Wulanchabu, Shanwei, Heyuan, Zhaotong, and Anshun. We found significant spatial autocorrelations for the city-level GVI, according to Moran’s *I* test (z-score: 6.95).

At the city level, the average GVI for 131 cities was 0.276, and the average standard deviation was 0.128. All cities were classified into four types according to the two values (Fig 5B). A high average and low standard deviation indicate a city whose street-level GVI was good and uniformly distributed in the city. There were 18 High Average Low StdD cities among all 131 cities. The top five were Weifang, Jiaxing, Zhuhai, Jiuquan, and Guangzhou. They are distributed widely in China. There were 46 cities associated with a low average and low standard deviation, indicating that the central areas of those cities were not green. They were also not limited to northern China as we had expected. Among the 43 high average and high standard deviation cities, streets were generally green but not uniformly distributed. Accordingly, the 24 low average and high standard deviation cities had similar conditions, but their overall conditions were not good.

We used linear regression to analyze the factors influencing the GVI of each city. These factors included LEVEL (administrative level: 1 for municipalities, 2 for subprovincial cities, 3 for other provincial capital cities, and 4 for prefecture-level cities) which is a continuous and categorical variable, SIZE (city size in terms of the total urban area), ECONOMY (economic production value), DESIGN (road junction density), DENSITY (population density in urban areas), ELEVATION (the average elevation above the sea level in meter), WATER (whether the central area of a city is crossed by any water body or not), MIDDLE (middle region of China or not), WEST (western region of China or not), TEMPERATURE (average temperature in degrees Celsius), HUMIDITY (average humidity), SUN (sunny hours), PRECIPITATION (precipitation in mm) and RAIN (raining days). The city attribute values were for the whole city; we were not able to extract the values for our study area (downtown) due to data limitations in yearbooks. We had to regard the downtown area as representative of the whole city. Potential biases, especially for the variables ECONOMY and DENSITY, may exist. The values for these variables came from statistical yearbooks. We conducted two tests to examine global autocorrelation using Moran’s I for both city-level and block-level GVI values, and did not find significant spatial autocorrelations. We found that only the variable WEST was positively significant, indicating that cities in western China tended to be associated with greener streets. This situation is contrary to the natural environment of western China, which is generally dry and has little precipitation. We assume that residents and decision makers in those cities have a greater preference for greenery than their counterparts in eastern China, and thus more effort is put into street greenery. We also found that the development and administrative levels of a city did not influence street greenery.

### 5.3 Intracity pattern analysis

There were 163,853 street segments and 173,425 locations in the 131 valid cities. The total length of these street segments was 24,267.6 km (average: 148.1 m). There were 47,748 blocks in these cities. The detailed statistical analysis is shown in Table 2. For all three forms of spatial units, over 80% had a GVI of less than 0.4 (not green or less green).

**Table 2:** Statistical descriptions for locations, street segments, and blocks in the street GVI for the 131 valid cities.

Type	No. of Features	Min.	Max.	Mean	Green View Index			
					< 0.2	0.2–0.4	0.4–0.5	> 0.5
Locations	173,425	0.000	0.913	0.277	55,962 (32.3%)	85,702 (49.4%)	21,224 (12.2%)	10,537 (6.1%)
Street segments with over 13 locations per km)*	23,917	0.002	0.840	0.261	8,188 (34.2%)	12,619 (52.8%)	2,258 (9.4%)	852 (3.6%)
Blocks greater than 1 ha with over 1 location per ha**	9,424	0.002	0.737	0.265	2,583 (27.5%)	5,931 (62.9%)	718 (7.6%)	192 (2.0%)

* “13” was the average location density value for all street segments.

** “1” was the average location density value for all blocks greater than 1 ha.

**Fig 6:**
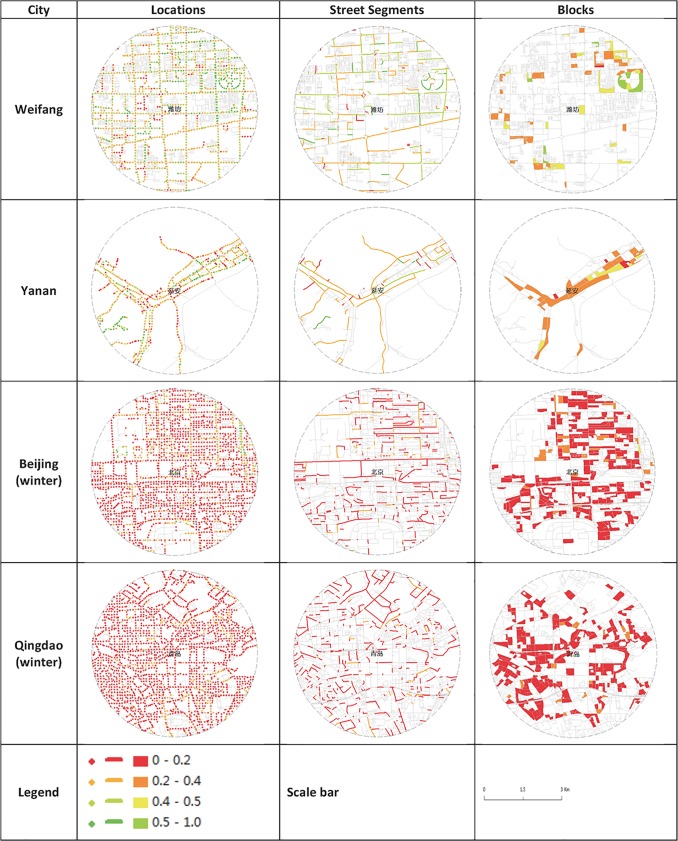
Street GVI for typical cities. Note that only street segments and blocks that follow the definition in Table 2 are mapped in the figure.

The street GVI patterns in the form of locations, street segments, and blocks are presented in Fig 6 (limited to typical cities due to space limitations). The patterns reveal the diversity and heterogeneity of cities. We analyzed the results based on the three respective aspects.

We explained the GVI of each street segment with more than 13 locations per kilometer in the valid cities with distinguished variables, including the air distance to the city center in meters (CENTER), street length in meters (LENGTH), and the control variables of the city where the street is. These variables are selected according to both the data availability and the domain knowledge as well as common sense of authors. Note all these explaining variables are not correlated with each other according to correlation analysis. We set three models for gaining knowledge on the impacting factors of location level GVI. As shown in Table 3, Model 1 only considers street level attributes, Model 2 includes city-level attributes, and Model 3 further adds city-level attributes to indicate where each city is in China. The regression results in Table 3 show that the more distant a street was from the city center, the greener it was. Longer streets in more economically developed and highly administrated cities tended to be greener. In addition, locations in middle and western China tended to be greener.

**Table 3:** Regression results for location-level street GVI.

Variables	Model 1		Model 2		Model 3	
	Coefficients	*p*-values	Coefficients	*p*-values	Coefficients	*p*-values
(Constant)						
**CENTER**	**0.051**	0.000	**0.048**	0.000	**0.047**	0.000
**LENGTH**	**0.106**	0.000	**0.115**	0.000	**0.115**	0.000
**SIZE**			-0.003	0.444	0.004	0.341
**LEVEL**			**-0.051**	0.000	**−0.036**	0.000
**DENSITY**			**-0.030**	0.000	**−0.042**	0.000
**ECONOMY**			**0.017**	0.000	**0.034**	0.000
**ELEVATION**			**-0.075**	0.000	**-0.153**	0.000
**WATER**			**-0.017**	0.000	**-0.032**	0.000
**MIDDLE**					**0.018**	0.000
**WEST**					**0.143**	0.000
Adjusted *R*^2^	0.014		0.025		0.036	

Note: coefficients in bold are significant at the 0.05 level. All coefficients for the three models have been standardized. The explanation of each variable is available in Section 5.2.

For distinguishing the impacting factors of different administrative level of cities, we have run Model 3 in Table 3 for each level of cities and the results are available in Table 4. Backward type linear regressions are employed in the models by level. The impact on factors like LENGTH, ELEVATION and WATER are consistent among all these models. However, we notice that this condition does not apply to some factors. For instance, the coefficient of CENTER for other provincial capital cities is on the contrary with that of other levels of cities and all cities. This requires more in-depth exploration in our future studies.

**Table 4:** Regression results for location-level street GVI for each level of cities.

Variables	All levels		LEVEL = 2		LEVEL = 3		LEVEL = 4	
	Coefficients	*p*	Coefficients	*p*	Coefficients	*p*	Coefficients	*p*
(Constant)								
**CENTER**	0.047	0.000	0.062	0.000	-0.040	0.000	0.053	0.000
**LENGTH**	0.115	0.000	0.094	0.000	0.090	0.000	0.117	0.000
**SIZE**					1.525	0.000	-0.011	0.001
**DENSITY**	−0.042	0.000			1.097	0.000	-0.041	0.000
**ECONOMY**	0.034	0.000			-0.338	0.000	0.011	0.001
**ELEVATION**	-0.153	0.000					-0.159	0.000
**WATER**	-0.032	0.000	-0.100	0.000	-0.751	0.000	-0.026	0.000
**MIDDLE**	0.018	0.000			-0.541	0.000		
**WEST**	0.143	0.000			-0.093	0.000	0.132	0.000
Adjusted *R*^2^	0.036		0.019		0.092		0.037	
N	173,425		3,636		12,141		157,648	

Note: all coefficients for the three models have been standardized. The explanation of each variable is available in Section 5.2.

We put all the streets in the 245 cities online; the street-based results can be viewed at http://geohey.com/app/dataviz/357b07615c4b4e25b76dcdd1ca9cd8f2/share?ak=ZmYzNmY0ZWJhYjcwNGU2ZGExNDgxMWUxNmZiOWNhNGY.

We also calculated the urban block level street GVI based on the streets surrounding a block. As a key quality-of-life component, this is expected to be a new indicator for planning control and urban management.

### 5.4 Validating the evaluation results

We manually calculated the GVI for 100 randomly selected SVPs in Photoshop. The correlation between the Photoshop-derived GVIs and those obtained through our study was 0.87, indicating an acceptable GVI calculation outcome.

In addition, we checked our street GVI rankings at the city level against a list of approved National Garden Cities (see http://baike.baidu.com/link?url=61VlWhMe353QmTKPJs86JGycywH8b4BxFewQmzW_iHVVglCJrts1x4AEtxgs42yYQqSNFvMJHk9dFg9rPRj-v_#5), which are evaluated by China’s central government, with GVI regarded as one of the most important indicators. The top five green cities in our rankings all appear on the list, and the lowest five are all absent.

## 6. Conclusion and discussion

### 6.1 Concluding remarks

In this study, we developed a methodological framework for analyzing visual greenery on streets using street-view pictures, which are widely available, even in developing countries. We expected to grasp street GVI using the framework, which included four procedures for achieving the target. First, we simplified detailed street segments to derive the locations to be used in the second step. Second, the API provided by Tencent Maps was used to gather and download SVPs with the necessary parameter settings for each location on a simplified street segment. For each location, we crawled four SVPs according to the direction of the street segment where the location was positioned (front, back, left, and right). Third, the color composition of each crawled SVP was calculated using MATLAB. The GVI of each SVP was derived using a predefined green range. The GVI of each location was calculated by aggregating the four SVPs at the location. Lastly, the location-level analysis results were aggregated at the street-segment and block levels to better understand street GVI in cities. We further analyzed street GVI at the city level. It should be mentioned that there were no privacy issues in this study since map providers address such issues [34].

The framework for analyzing street GVI was applied to the downtown areas (28.3 km^2^ each) of 245 major Chinese cities, falling into the pool of the Big Model paradigm proposed by [35]. Over one million SVPs were collected for the 336,990 locations on the streets. In addition to results validation, we analyzed these calculation results at both the intercity and intracity levels. Our main findings are as follows: (1) A total of 131 cities (336,990 locations) were used for street analysis since some SVPs in the other 114 cities were taken during autumn or winter, making them unsuitable for analyzing street GVI. (2) The cities Weifang, Zigong, Baoji, Maanshan, and Chengde had the best street GVI, and Wulanchabu, Shanwei, Heyuan, Zhaotong, and Anshun had the worst. (3) Cities in western China tended to have greener streets. (4) Longer streets on small blocks in more economically developed, highly administrated, less populated cities tended to be greener.

### 6.2 Potential applications

Each location, street segment, and block in the downtown areas of 245 major Chinese cities was associated with a GVI after applying our proposed framework. This provides various stakeholders with a new set of data for understanding street greenery and how it varies across a city and between cities. The potential applications include, but are not limited to, the following: **First**, the greenness of a street or block can be employed as a new indicator for street walkability evaluation, real estate pricing, and even road navigation. This can facilitate better-informed urban design. **Second**, the results can inform urban landscape and gardening planning/design in locating interesting sites for improvement. **Third**, the greenness surrounding a block can be combined with the green cover control of a block (commonly used in zoning in the USA and detailed planning in China) in urban planning and management to provide enough green space and street greenery for citizens.

### 6.3 Academic contributions

In addition to its potential applications in urban practice, this study makes the following academic contributions (Table 5 compares our method with that of [4]). **First**, the proposed framework, based on [4–5] with modifications, was applied to a large number of cities. In contrast to existing studies, such as [3–5], which were limited to a single city, our large-scale analysis allowed us to detect universal laws of street GVI. Research on visual greenery has long been confined to small samples because of the manual and subjective nature of the evaluation process. Our proposed approach is expected to push the area forward toward more duplicable methods. **Second**, we paid more attention to the spatial patterns of GVI at various geographical units (e.g., sites, streets, and blocks), thus revealing the diversity and heterogeneity of urban greenery. **Third**, we used regression analysis to gain knowledge about the factors potentially affecting street greenery at both the intracity and intercity levels. This may inform urban planning and design practice.

**Table 5:** Our method versus that of Li et al. [4].

Comparison Dimension	This Study’s Method	[4]
Sampling strategy	Every 50 m on streets	Random
Data source used	Tencent	Google
Validation	Photoshop, SegNet, and official list of National Garden Cities	Photoshop
Application area	Central areas of 245 cities	A local area of a city
Spatial unit(s) for analysis	Site, streets, and blocks	Sites
City-level comparison	Yes	No
Results derived	Both intra- and inter-city findings	Intracity findings

### 6.4 Limitations and future research

There are a number of areas that warrant more attention in future research. **First**, there is room to improve the specifications of the current study (e.g., the horizontal and vertical angle settings, the distance interval for crawling SVPs, and the color-range setting representing greenness). **Second**, we should extend the study area to all urban areas in all major Chinese cities to gain a more holistic view of street greenery in China, which would make it possible to compare greenness between downtown and suburban areas. **Third**, we are willing to expand our perspective from street greenness to other aspects that SVPs are able to show. These include the diversity and consistency of street color, social and commercial activity and vibrancy, the degree of physical order/disorder on a street, infrastructure for active travel, pedestrian safety, motorized traffic and parking, and the aesthetics of regional expressways. **Last but not least**, given that some sites have the same GVI but look quite different (some sites are fancy while others are not), SVPs reflecting objective street landscapes can be integrated with subjective geotagged and user-contributed photos to better understand the street space of a city.
